# PEAR1 Promotes Myoblast Proliferation Through Notch Signaling Pathway

**DOI:** 10.3390/biology13121063

**Published:** 2024-12-19

**Authors:** Yahao Zhao, Lu Zhang, Ruotong Hao, Shuang Li, Shufeng Li, Shuai Shi, Huili Tong, Bingchen Liu

**Affiliations:** 1 Key Laboratory of Animal Cellular and Genetics Engineering of Heilongjiang Province, Northeast Agricultural University, Harbin 150030, China; zyhbiology@gmail.com (Y.Z.); 15528667696@163.com (L.Z.); hao18845756435@163.com (R.H.); lishuang8927@neau.edu.cn (S.L.); lishufeng1@neau.edu.cn (S.L.); 2 Laboratory of Cell and Developmental Biology, Northeast Agricultural University, Harbin 150030, China; shishuai@hrbmu.edu.cn; 3 Department of Cardiology, The Fourth Affiliated Hospital of Harbin Medical University, Harbin 150006, China; 4 Department of Cardiology, The Second Affiliated Hospital, School of Medicine, Zhejiang University, Hangzhou 310009, China; 5 State Key Laboratory of Transvascular Implantation Devices, Hangzhou 310009, China; 6 Heart Regeneration and Repair Key Laboratory of Zhejiang Province, Hangzhou 310009, China

**Keywords:** PEAR1, Notch signaling pathway, myoblast proliferation, skeletal muscle regeneration

## Abstract

PEAR1, also known as platelet endothelial aggregation receptor 1, is known to play a crucial role in the migration and differentiation of muscle satellite cells (MuSCs). However, its specific effects on skeletal muscle development and regeneration require further exploration. In this study, the expression of PEAR1; the proliferation marker proteins of Pax7, CCNB1, and PCNA; and the key molecules of N1-ICD, N2-ICD, and Hes1 were all increased gradually during the process of C2C12 cell proliferation. Furthermore, Western blotting and EdU results showed that when PEAR1 was over-expressed or inhibited, the proliferation status of C2C12 cell was increased or reduced respectively. This implied that PEAR1 could regulate myoblast proliferation and might be relate to Notch cell signaling pathway. A subsequent immunoprecipitation experiment result showed that the interaction between PEAR1 and Notch1 or Notch2, respectively. Then Western blotting and EdU results showed that the proliferation of C2C12 cell was inhibited under the treatment of Notch signaling pathway inhibitor RIN1. Meanwhile, the proliferation capacity of C2C12 cell could not be improved by treatment with RIN1 even though PEAR1 was over-expressed. These results showed that PEAR1 may regulated C2C12 cell proliferation though Notch signaling pathway. Additionally, a mouse model of muscle injury repair injected with bupivacaine hydrochloride was established in this study. Immunohistochemistry results exhibited that PEAR1 may regulate skeletal muscle post-injury regeneration relevant to Notch1 and Notch2 in different patterns. These findings provide valuable insights into the potential involvement of PEAR1 in skeletal muscle development and post-injury regeneration.

## Simple Summary

This study demonstrated that PEAR1 can act as a novel receptor for the Notch signaling pathway and regulate myoblast proliferation by using C2C12cells and establishing a mouse skeletal muscle injury repair animal model. These findings contribute to the repair and regeneration of skeletal muscle injuries, providing new solutions for skeletal muscle aging, injury, and atrophy.

## 1. Introduction

Skeletal muscles, as one of the important tissues in the human body, undertake various important life activities such as movement, metabolism, and respiration. The development and regeneration of skeletal muscle depend on muscle satellite cells (MuSCs). MuSCs, as a type of myoblast stem cells, respond to the stimulate factors to activate, proliferate, migrate and differentiate into new muscle fibers [1]. This is very important for skeletal muscle development and regeneration. This process is influenced by various factors, including the regulation of endogenous gene expression, such as the myogenic regulatory factors (MRFs) family and other functional genes, as well as other types of cells (fibro-adipogenic progenitor cells, pericytes, macrophages and etc.), extracellular matrix, and nutrients (such as vitamin C) [2,3,4]. In summary, the regeneration of MuSCs into new muscle fibers is an extremely complex and highly precise biological process. Clarifying the molecular mechanisms of this process can provide important support for the treatment of skeletal muscle-related diseases and the improvement of human health.

Platelet Endothelial Aggregation Receptor 1 (PEAR1, also known as JEDI or MEGF12), is a type I transmembrane protein named after its high expression in platelets and vascular endothelial cells and its involvement in platelet aggregation. Since its discovery, research on PEAR1 has mostly focused on the production and aggregation of blood platelets as well as on angiogenesis. PEAR1, MEGF10, and MEGF11 belong to the multi-epidermal-growth-factor-like protein family, and members of this family have similar extracellular domains with multiple EGF-like repeating domains [5,6]. The extracellular portion of the mouse PEAR1 protein contains fifteen EGF repeats and a DSL structural domain, while the intracellular region contains five proline-rich structural domains [7].

Although the role of MEGF10, a member of the PEAR1 family, in skeletal muscle development is well known, it has been confirmed that MEGF10 can participate as a Notch1 ligand in regulating muscle cell proliferation [8]. There are few reports on PEAR1 in skeletal muscle development. A previous report by our team indicated that PEAR1 is involved in C2C12 cell differentiation and may be involved in the process of skeletal muscle injury and repair [9]; meanwhile, PEAR1 promoted the migration and differentiation of bovine MuSCs by interacting with integrins β in bovine MuSCs [10]. There have been no studies exploring the roles of PEAR1 on myoblast proliferation. In this study, we found that PEAR1 may play a new and important role in regulating C2C12 cell proliferation in vitro, and PEAR1 may serve as a new ligand for the Notch signaling pathway to regulate mouse myoblast proliferation in vitro and in vivo.

The proliferation process of MuSCs is regulated by various regulatory factors and signaling pathways, and the Notch signaling pathway is one of the important signaling pathways that regulate MuSCs proliferation. The Notch signaling pathway is a conserved signaling pathway in mammals, which includes Notch ligands (Jagged1, Jagged2, Dll1, Dll3, Dll4), Notch receptors (Notch1, Notch2, Notch3, Notch4), and downstream effector molecules of the Notch signaling pathway (such as Hes1, Hey1, HeyL). After Notch ligands bind to receptors, γ-secretase enzymes cleave the Notch intracellular domain (NICD) from the Notch receptor. After cleavage by γ-secretase enzymes, NICD can freely migrate to the nucleus. NICD enters the nucleus and interacts with the DNA-binding protein RBPJ, subsequently causing upregulation of downstream effector molecules in the Notch signaling pathway [11]. The Notch signaling pathway regulates the growth and development status of various tissues and stem cells and plays an important role in the growth and development of skeletal muscles [12,13].

C2C12 cells and mouse skeletal muscle injury repair animal models were used in this study to elucidate the molecular mechanism of PEAR1 as a new receptor for the Notch signaling pathway, then regulating myoblast proliferation. These findings aim to provide theoretical support for maintaining the homeostasis balance of skeletal muscle tissue as well as to offer reliable insights and data support for muscle health issues, such as skeletal muscle regeneration, aging, and sarcopenia.

## 2. Materials and Methods

### 2.1. Animal Experimental and Ethics Statement

The experimental animals, female mice, were purchased from YISI in Changchun under the supervision of the Animal Protection Committee of the Northeast Agricultural University. This study complies with the requirements of the ethical system for experimental animals and follows the principle of treating animals well to ensure animal welfare. The approval number for ethical review is NEAUEC20210112. The mice were grown in an artificially simulated environment with an equal-length day–night cycle and free access to food and water. A drug injection experiment was performed to induce skeletal muscle injury when the mice’s weight reached 25 g. Ether was used to anesthetize the mice, and 50 μL of bupivacaine hydrochloride solution (with a concentration of 0.5%) was injected into the tibialis anterior (TA) muscle of the mice’s lower limbs. The TA muscles were collected on days 0, 1, 3, 5, 7, and 14, respectively, after the injury.

### 2.2. Cell Culture

C2C12 cells (Procell, Wuhan, China) were cultured in DMEM high-sugar medium (Gibco, Grand Island, NY, USA) containing 10% fetal bovine serum (Biological Industries, Beit Haemek, Israel) (growth medium) at 37 °C in a constant temperature incubator with 5% CO_2_. Cells were grown to a density of approximately 80% and digested with 0.25% trypsin for passage. Then, C2C12 cells were cultured for continuous proliferation in growth medium at 12 h, 24 h, and 48 h respectively. Meanwhile, the cells for transfection, co-immunoprecipitation, EdU assay, and flow cytometry analysis in this study were all cultured in growth medium for proliferation. It should be noted that all the cell samples were collected when the density reaches approximately 80% to avoid contact inhibition of cell growth.

### 2.3. Overexpression and Inhibition of PEAR1

Primers of 5′-GTGCTGTTTTGTTACCGTG-3′ and 5′-AAGTGTTTCTGTTCGTGGC-3′ were designed to amplify the CDS sequence of PEAR1, then the PEAR1 CDS sequence (GenBank: BC042490.1) was cloned into the empty vector of pcDNA3.1+ to construct pcDNA3.1-PEAR1 (designated as PEAR1-OE). Additionally, shRNA interference sequence targeting PEAR1 was synthesized, annealed and ligated into the vector of pRANT-H1.1/Shuttle-RFP to inhibit the expression of PEAR1 (designated as PEAR1-shRNA). The shRNA sequence was designed as follows: 5′-GCCATATCCCTGGACACTATGCGAACATAGTGTCCAGGGATATGGCTTTTTT-3′ and 5′-AAAAAAGCCATATCCCTGGACACTATGTTCGCATAGTGTCCAGGGATATGGC-3′. PEAR1-OE or PEAR1-shRNA was transfected into C2C12 cells to overexpress or inhibit the expression of PEAR1 separately in subsequent experiments.

C2C12 cells were seeded in 6-well plates. When the cell density reached approximately 80%, PEAR1-OE or PEAR1-shRNA was transfected into the cells using Lipofectamine 2000 (11668027, Thermo Fisher Scientific, Waltham, MA, USA) according to the manufacturer’s protocol.

### 2.4. Western Blotting

The protein samples extracted were mixed with loading buffer (P0015L Beyotime Biotechnology, Shanghai, China) and then boiled in a water bath for 10 mins. Subsequently, SDS-PAGE gel electrophoresis (EC0023-B SparkJade, Jinan, China) was performed to separate the proteins, followed by their transfer onto PVDF membranes (Millipore, Billerica, MA, USA). The membranes were then blocked with 10% skimmed milk powder at 37 °C for 1 h. Next, the membranes were incubated overnight at 4 °C with specific primary antibodies, including PEAR1 antibody (sc-292937, Santa Cruz Biotechnology, Dallas, TX, USA), Notch1 antibody (bs1335R, Bioss, Beijing, China), Notch2 antibody (WL02409, WanLeibio, Shenyang, China), Pax7 antibody (bs-2413R, Bioss, China), CCNB1 antibody (A2056, ABclonal, Wuhan, China), PCNA antibody (bs-2007R, Bioss, China), Hes1 antibody (bs-2972R, Bioss, China), and GAPDH antibody (bs-0755R, Bioss, China). After incubation, the membranes were washed four times with PBST buffer for 8 mins each time. Subsequently, the membranes were incubated with secondary antibodies (bs-80295G-HRP, Bioss, China) at 37 °C for 1 h, followed by another four washes with PBST for 8 mins each. Finally, chemiluminescence was used for color development, and the PVDF membranes were photographed with a MiniChemi™ 500 Mini Chemiluminescent Imaging and Analysis System (Sage Creation Science, Beijing, China).

### 2.5. Co-Immunoprecipitation

PEAR1 antibody and magnetic beads (HY-K0202, MCE, Monmouth Junction, NJ, USA) were incubated together for 2 h at 4 °C in the IP group, while rabbit IgG (HY-K0202, Beyotime, Shanghai, China) and magnetic beads were incubated together for 2 h at 4 °C in the IgG group. The magnetic beads were then incubated with the extracted total cellular protein for 2 h at 4 °C, and the liquid was discarded using a magnetic rack to retain the beads. The supernatant was collected in a new tube, and Western blotting was performed to detect the presence of reciprocal binding between PEAR1 and Notch1 as well as Notch2. Subsequently, incubation with Notch1 antibodies, Notch2 antibodies, and magnetic beads was conducted to verify the reciprocal binding of Notch1 or Notch2 with PEAR1.

### 2.6. RIN1 Treatment

RBPJ Inhibitor-1 (RIN1) (Catalog Number: HY-137471), which purchased from MedChemExpress (Shanghai, China), blocks the function of RBPJ to inhibit the Notch signaling pathway. RIN1 was diluted with DMSO and its final concentration for use was 0.6 μM, according to the reference [14]. Meanwhile, an equal amount of DMSO was added into cell culture medium as the control group. Then C2C12 cells were cultured with or without RIN1 (control group) and collected for subsequent experiments.

### 2.7. EdU Assay

EdU reagent (5-Ethynyl-2′-deoxyuridine) (C0078S) was purchased from Beyotime Biotechnology, Shanghai, China. C2C12 cells were transfected or treated with RIN1 inhibitors. Two hours before the end of the treatment, EdU was added to the cell culture medium and incubated for 2 h. The cells were then fixed with 4% paraformaldehyde for 15 min, permeabilized with PBS containing 0.3% Triton X-100, and subjected to a click reaction. Nuclei were stained with Hoechst 33,342 solution and mounted using an AntiFade Mounting Medium (P0131, Beyotime Biotechnology, Shanghai, China) before observation under a fluorescent microscope.

### 2.8. Flow Cytometry Analysis

Flow cytometry analysis was used in this study to assess the changes of C2C12 cell cycle influenced by PEAR1 overexpressed or inhibited. A Cell Cycle Analysis Kit (C1052, Beyotime Biotechnology, Shanghai, China) was used in this experiment. The procedure was performed according to the instructions provided in the product manual. The simple procedure involved cell sample preparation, PI staining, incubation, flow cytometry analysis, data analysis using a Cytomics FC500 Flow Cytometer (Beckman Coulter, Fullerton, CA, USA), and analysis with CXP Software ver.2.3 (Beckman Coulter).

### 2.9. HE Staining

HE staining was performed using Hematoxylin-Eosin (HE) Stain Kit (Catalog Number: G1120, Solarbio Life Sciences, Beijing, China). Paraffin sections were incubated at 37 °C for 16 h, dewaxed, stained with hematoxylin for 3 mins, differentiated, and observed under a microscope. Eosin staining was performed, followed by dehydration with graded alcohol and sealing with neutral gum. The sections were then photographed for further analysis.

### 2.10. Immunohistochemistry

Paraffin sections were dewaxed, subjected to antigen retrieval at 95 °C for 15 mins, and treated with hydrogen peroxide solution. After blocking with horse serum, the sections were incubated overnight with primary antibodies at 4 °C. Then, secondary antibodies were applied, and DAB (ZLI-9018, ZSGB-BIO, Beijing, China) was used for color development. Nuclei were counterstained with hematoxylin. The sections were then photographed using an Upright Microscope (BX53, Olympus, Tokyo, Japan).

### 2.11. Statistical Analysis

The experiments in this study were repeated at least three times. The data were expressed as mean ± standard error of the mean. The grayscale values of Western blotting bands were scanned by Image J software (https://imagej.net/ij/, accessed on 16 October 2024). GraphPad Prism 7 (https://www.graphpad-prism.cn/, accessed on 16 October 2024) was used for all data analysis in the study. The statistical method for comparing corresponding two groups was Student’s *t*-test; a significance level of 0.05 was used to determine statistical significance, denoted by “*” for *p* < 0.05, indicating significant difference, and “**” for *p* < 0.01, indicating extremely significant difference. One-way ANOVA analysis was used for multi-group comparisons. The different letters a, b c, and d represent significant differences between different groups, while the same letters represent no significant differences between different treatment groups.

## 3. Results

### 3.1. The Expression Pattern of PEAR1 and Notch Signaling Pathway During C2C12 Cell Proliferation

To investigate the role of PEAR1 related to C2C12 cell proliferation and the Notch signaling pathway, Western blotting was used to detect the expression of PEAR1; Pax7, CCNB1, PCNA (Related to cell proliferation); Notch1, Notch2, Hes1, N1-ICD, and N2-ICD (related to the Notch signaling pathway).

**Figure 1:**
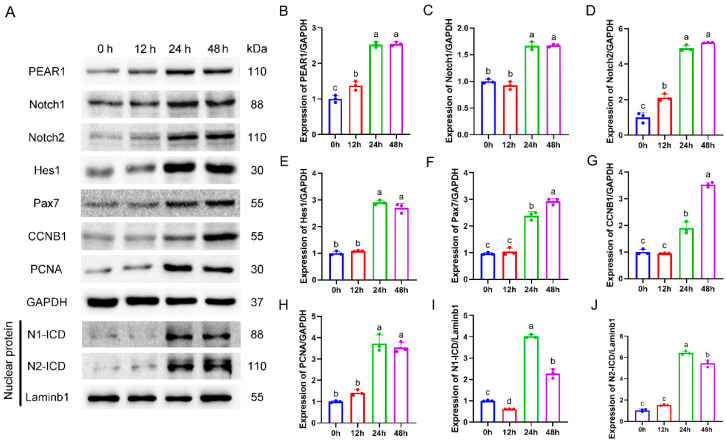
**The expression pattern of PEAR1 and Notch signaling pathway during C2C12 cell proliferation.** (**A**) Western blotting results of PEAR1, Pax7, CCNB1, PCNA and marker proteins related to the Notch signaling pathway. (**B**–**J**). Statistical data based on A. Laminb1 is a nuclear reference protein for Western blotting detection. The different letters a, b, c, and d represent significant differences between different groups, while the same letters represent no significant differences between different treatment groups. The original Western Blot images can be found in the Supplementary File.

The expression levels of PEAR1 and Pax7 both increased gradually during the process of C2C12 cell proliferation (From 0 h to 48 h). Meanwhile, the expression levels of the proliferation marker proteins CCNB1 and PCNA all increased accompanied by the progression of C2C12 cell proliferation. Moreover, the expression levels of N1-ICD and N2-ICD in the nucleus both significantly increased. In addition, the expression levels of Hes1, downstream key molecule of Notch signaling pathway also increased (Figure 1A–J). The expression levels of PEAR1, Pax7, CCNB1, PCNA, Notch1, Notch2, Hes1, N1-ICD, and N2-ICD at 48 h was all increased more than 2-fold, 1-fold, 4-fold, 3-fold, 2-fold, 3-fold, 4-fold, 3-fold, and 6-fold, respectively compared to their corresponding groups at 0 h. These results indicated that the expression of PEAR1 and the Notch signaling pathway may be relevant to C2C12 cell proliferation.

### 3.2. PEAR1 Regulates C2C12 Cell Proliferation and Notch Cell Signaling Pathway

**Figure 2:**
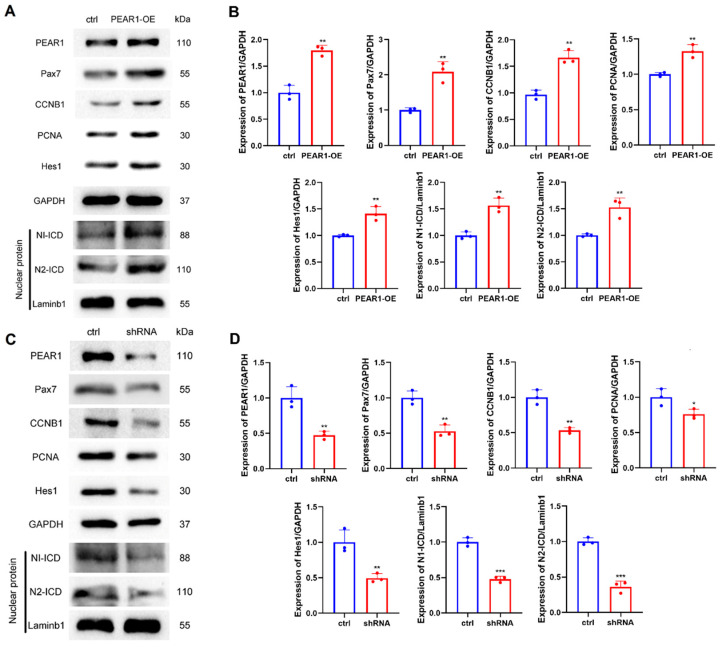
PEAR1 regulates the expression of proliferative marker proteins and Notch cell signaling during C2C12 cell proliferation. (**A**) Western blotting results of the expression of Pax7, CCNB1, PCNA, and Notch signaling pathway marker proteins after PEAR1 over-expression; ctrl represents the control group (pcDNA3.1+ empty vector); PEAR1-OE represents the PEAR1 over-expression group. Laminb1 is a nuclear reference protein for Western blotting detection. (**B**) Statistical data based on A. (**C**) Western blotting results of the expression of Pax7, CCNB1, PCNA, and Notch signaling pathway marker proteins after PEAR1 was inhibited; ctrl represents the shRNA negative control group; shRNA represents the PEAR1 inhibition group. (**D**) Statistical data based on C. “*” for *p* < 0.05, indicating significant difference, “**” for *p* < 0.01, indicating extremely significant difference, and “***” for *p* < 0.001, indicating extremely significant difference. The original Western Blot images can be found in the Supplementary File.

PEAR1 was up-regulated and down-regulated to explore its function in regulating C2C12 cell proliferation. Western blotting results showed that the expression of proliferative marker proteins of Pax7, CCNB1, and PCNA were all significantly up-regulated or down-regulated when PEAR1 was over-expressed or inhibited at 48 h. Furthermore, the expressions of N1-ICD and N2-ICD in nuclei were significantly up-regulated with PEAR1 over-expression. As expected, the expressions of N1-ICD and N2-ICD in nuclei were all down-regulated at 48 h when PEAR1 was inhibited by shRNA interference (Figure 2).

**Figure 3:**
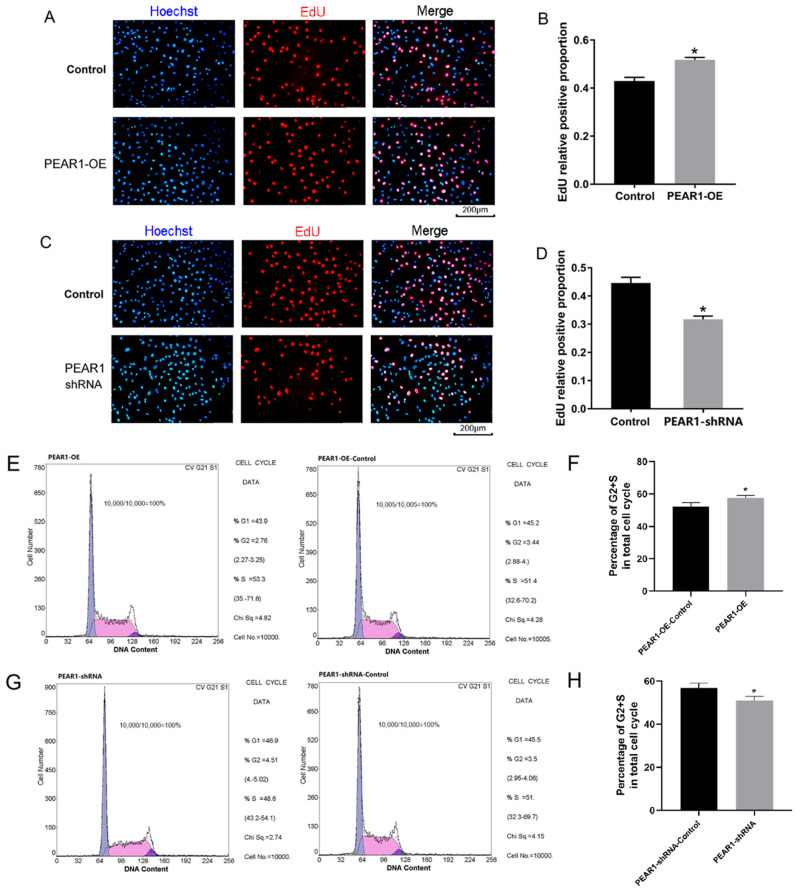
**PEAR1 regulates C2C12 cell proliferation.** (**A**,**C**) EdU results after PEAR1 over–expressed or inhibited in C2C12 cells. The blue signal represents for cell nuclei stained by Hoechst 33,342 and red signal represents EdU positive cells. (**B**,**D**) Statistical data based on (**A**,**C**), respectively. (**E**,**G**) Flow cytometry results after PEAR1 over–expressed or inhibited in C2C12 cells. (**F**,**H**) Statistical data based on (**E**,**G**), respectively. “*” for *p* < 0.05, indicating significant difference.

Meanwhile, EdU assay results showed that the EdU positive cells increased or decreased accompanied by PEAR1 over-expressed or inhibited at 48 h (Figure 3A–D). Similar results were obtained by flow cytometry analysis and the results showed that the overexpression or inhibition of PEAR1 could promote or hinder the progression of C2C12 cell proliferation (Figure 3E–H). In summary, these results indicated that PEAR1 regulates C2C12 cell proliferation and may be related to the Notch cell signaling pathway.

### 3.3. The Interaction Between PEAR1 and Notch1 or Notch2

**Figure 4:**
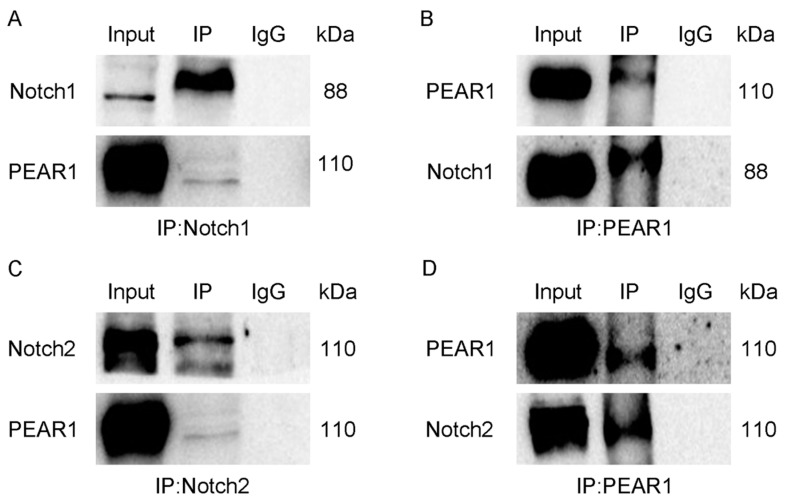
**Co-IP results of PEAR1 interacting with Notch1 or Notch2, respectively.** (**A**). Co-IP with Notch1 antibody followed by Western blotting using Notch1 and PEAR1 antibody. (**B**). Co-IP with PEAR1 antibody followed by Western blotting using PEAR1 and Notch1 antibody. (**C**). Co-IP with Notch2 antibody followed by Western blotting using Notch2 and PEAR1 antibody. (**D**). Co-IP with PEAR1 antibody followed by Western blotting using PEAR1 and Notch2 antibody. InPut represents positive control group, IgG represents negative control group, IP represents the target group. The original Western Blot images can be found in the Supplementary File.

In order to investigate the relationship between PEAR1 and Notch1 or Notch2, a Co-Immunoprecipitation (Co-IP) experiment was performed to verify the interaction between PEAR1 and Notch1 or Notch2 respectively. Immunoprecipitation results with anti-PEAR1 antibodies showed that Notch1 or Notch2 was expressed in the precipitated complex respectively. Meanwhile, PEAR1 was detected in the precipitated complexes of both anti-Notch1 and anti-Notch2 antibody immunoprecipitation groups separately (Figure 4). The results indicated that PEAR1 interacts with Notch1 or Notch2, respectively.

### 3.4. PEAR1 Regulates C2C12 Cell Proliferation Through Notch Signaling Pathway

**Figure 5:**
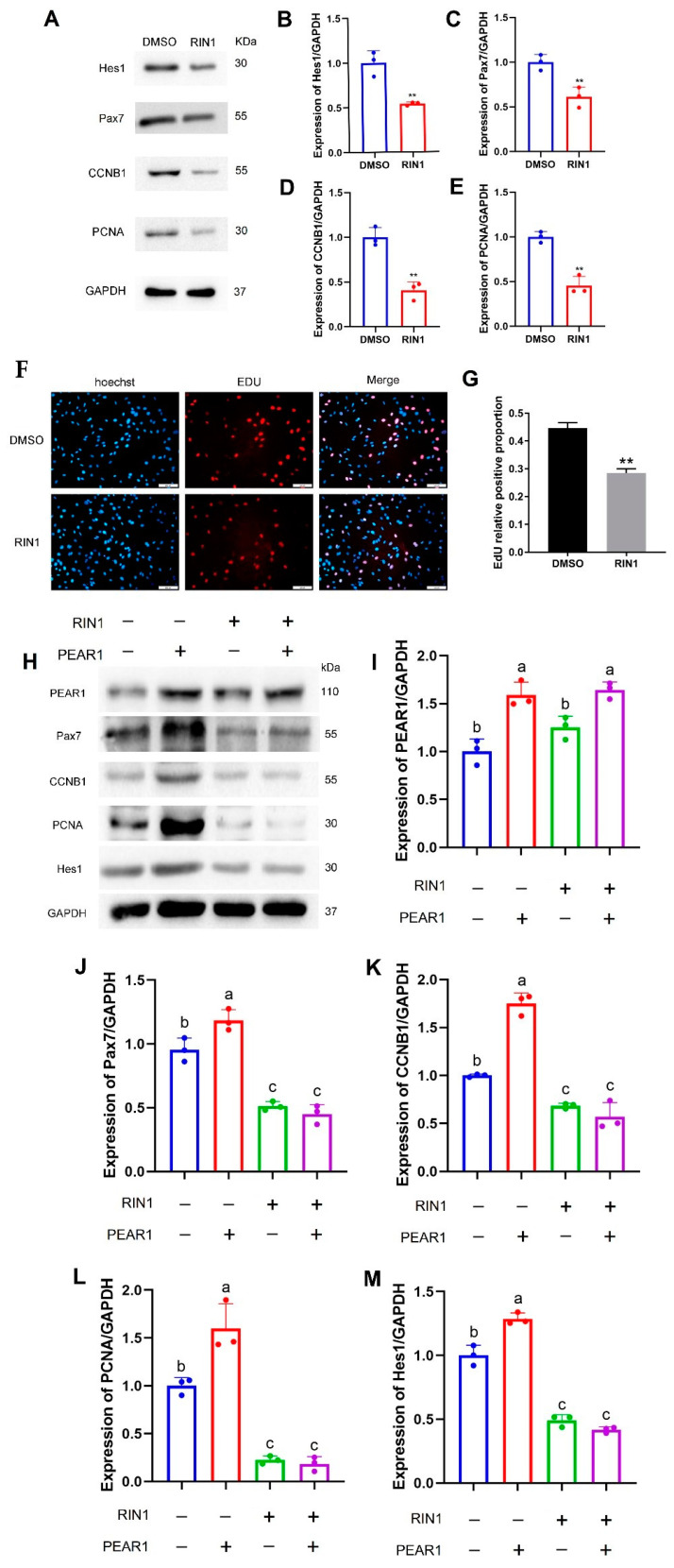
**PEAR1 regulates C2C12 cell proliferation through the Notch signaling pathway.** (**A**) Western blotting results of Hes1, Pax7, CCNB1, and PCNA. (**B**–**E**) Statistical data based on (**A**). (**F**) EdU results in C2C12 cells treated by RIN1. The blue signal represents for cell nuclei stained by Hoechst 33,342, and the red signal represents EdU-positive cells. (**G**). Statistical data based on (**F**). (**H**) Western blotting results of PEAR1, Pax7, CCNB1, PCNA, and Hes1. (**I**–**M**) Statistical data based on (**H**). “**” for *p* < 0.01, indicating extremely significant difference. Different letters of a, b and c represent significant differences between different groups, while the same letters represent no significant differences between different treatment groups. The original Western Blot images can be found in the Supplementary File.

A Notch signaling pathway inhibitor RIN1 (used at a concentration of 0.6 μM) was added into C2C12 cell culture medium. Western blotting results showed that the expression of Hes1 down-regulated significantly compared with the control group at 48 h treated with RIN1. This indicated Notch signaling pathway inhibited by RIN1. Due to this, the proliferation marker proteins of Pax7, CCNB1, and PCNA were all significantly down-regulated when Notch signaling pathway was inhibited (Figure 5A–E). Meanwhile, the EdU positive cells were also decreased by RIN1 treatment (Figure 5F,G).

In order to deeply investigate the relationship between PEAR1 and the Notch signaling pathway, PEAR1 was over-expressed in C2C12 cells then the expression of Pax7, CCNB1, PCNA, and Hes1 was up-regulated. However, when the PEAR1 overexpression vector was transfected into C2C12 cells and RIN1 was added into the cell culture medium at the same time. Western blotting results showed that the expression of Pax7, CCNB1, PCNA and Hes1 could not be up-regulated when the Notch signaling pathway inhibited by RIN1 even though PEAR1 was over-expressed (Figure 5H–M). These results suggested that PEAR1 regulates C2C12 cell proliferation mainly through the Notch signaling pathway.

### 3.5. The Expression Pattern of PEAR1 and Notch Signaling in Mice Skeletal Muscle Post-Injury Regeneration

**Figure 6:**
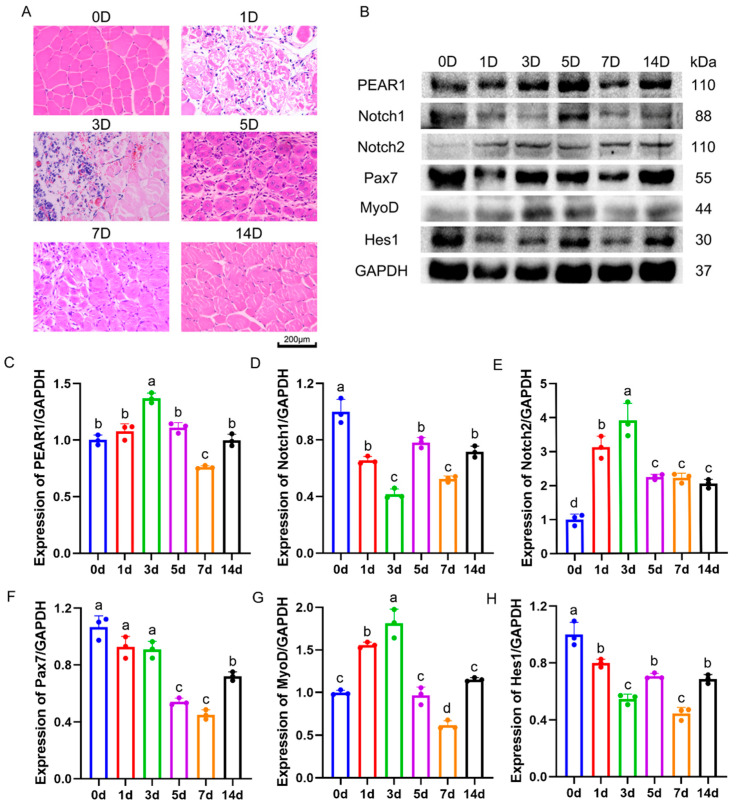
**Expression localization of PEAR1, Pax7, Notch1, and Notch2 during skeletal muscle post-injury regeneration.** (**A**). HE staining was used to identify whether the mouse muscle injury repair model was successfully constructed. The red part is stained with eosin, which gives the cytoplasm a red color. The blue color is hematoxylin staining, which gives the nucleus a blue color. The indicators 0 D, 1 D, 3 D, 5 D, 7 D, and 14 D indicate that muscle injury repair was carried out on Day 0, Day 1, Day 3, Day 5, Day 7, and Day 14. (**B**). Western blotting results of PEAR1 and Notch signaling pathway molecules in the process of post-injury regeneration. (**C**–**H**). The results of the analysis based on (**B**). The different letters a, b, c, and d represent significant differences between different groups, while the same letters represent no significant differences between different treatment groups. The original Western Blot images can be found in the Supplementary File.

A mice skeletal muscle post-injury regeneration animal model was constructed by injecting 0.5% bupivacaine hydrochloride into the tibialis anterior muscle. HE staining showed that muscle fiber injury started 1 day after the injection of bupivacaine hydrochloride, small, newly formed muscle fibers appeared on Day 3 after injury, and a large number of newly formed thick muscle fibers appeared on Day 5. The muscle completed regeneration by Day 14 (Figure 6A). The expressions of PEAR1, Notch1, Notch2, Pax7, MyoD, and Hes1 were detected using Western blotting during the process of post-injury regeneration. Pax7 was expressed at high levels from Day 1 to Day 3, then declined from Day 5 to Day 7 and showed a recovery trend by Day 14. The expression of MyoD continued to increase from Day 1 to Day 3, then decreased from Day 5 to Day 7 and increased again by Day 14 (Figure 6B–H).

**Figure 7:**
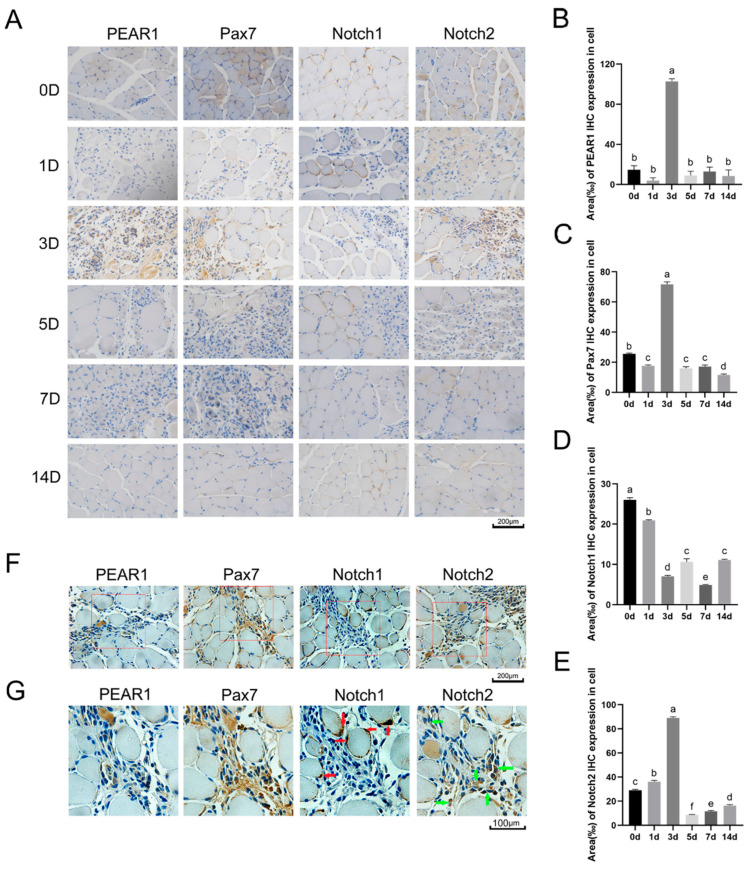
**Immunohistochemistry staining for PEAR1, Pax7, Notch1, and Notch2.** (**A**). Immunohistochemistry was used to detect the expression patterns of PEAR1, Pax7, Notch1, and Notch2 the skeletal muscle post-injury process. Regeneration brown signal refers to the staining of the target molecule by DAB, and blue refers to the staining of nucleus. The indicators 0 D, 1 D, 3 D, 5 D, 7 D, and 14 D indicate that skeletal muscle post-injury regeneration was carried out on Day 0, Day 1, Day 3, Day 5, Day 7, and Day 14. (**B**–**E**). The results of the analysis based on A.F. Immunohistochemistry was used to detect the expression and localization of PEAR1, Pax7, Notch1 and Notch2 on Day 3 after the injury. The same parts were photographed, and the picture content in the red box was selected for amplification. (**G**). Enlarged picture based on (**F**). Red arrows indicate the positive signals of Notch1, and green arrows indicate the positive signals of Notch2. The different letters a, b, c, d, e and f represent significant differences between different groups, while the same letters represent no significant differences between different treatment groups.

The expressions of PEAR1, Notch1, Notch2, and Pax7 were then measured by immunohistochemistry during the process of skeletal muscle post-injury regeneration. Serial tissue sections were used for staining. The results showed the positive signals for PEAR1, Notch2, and Pax7 during the injury repair process on Day 3, and Notch1 was expressed throughout the process. The positive signal for Notch1 was located at the edges of the muscle fibers, and some of the positive signals were co-localized with Pax7. These results suggested that PEAR1 was involved in the early stages of skeletal muscle post-injury (Day 1 to Day 3) (Figure 7A). It was interesting that the expression pattern of Notch1 and Notch2 was different. It implies that Notch1 and Notch2 may play different roles in skeletal muscle post-injury. Therefore, immunohistochemistry staining was carried out to investigate the locations of PEAR1, Notch1, and Notch2, respectively.

Pax7, as a maker of MuSCs, showed positive signals at the post-injury sites. It showed that the MuSCs rapidly proliferated when the skeleton suffered from injury. PEAR1 had the same expression location compared to Pax7 at Day 3 after skeletal muscle injury (Figure 7A–E). These results suggested that when the skeletal muscle injured, PEAR1 may up-regulate and promote MuSCs proliferation. In addition, the amplified images on Day 3 after the injury showed that Notch1 and Notch2 had different expression patterns. Notch2 was mainly located at the myofibers, while Notch1 was mainly located at the Pax7-positive area (Figure 7F,G). It supposed that PEAR1 may activate Notch signaling pathway to promote MuSCs proliferation with different patterns related to Notch1 and Notch2.

## 4. Discussion

Since the discovery of PEAR1, its functionality has garnered significant attention. Research indicates that PEAR1 is known in platelet aggregation and megakaryopoiesis [15,16]. For instance, PEAR1 influences imegakaryopoiesis and thrombopoiesis through phosphatase and tensin homolog (PTEN) to regulate the phosphatidylinositol 3-kinase (PI3K)-Akt and Notch signaling pathways [13]. In this study, we revealed the new significant role of PEAR1 in promoting myoblast proliferation. Pax7 serves as a marker for MuSCs, expressed in both quiescent satellite cells and proliferating myoblast [17]. Therefore, an elevated Pax7 expression indicated that C2C12 myoblast cells were stimulated to proliferate. Furthermore, both CyclinB1 and PCNA were involved in the cell cycle—the changes in their expression levels can also reflect the cell proliferation status [18,19]. Consequently, Western blotting results showed that Pax7, CyclinB1, and PCNA were all significantly up-regulated or down-regulated when PEAR1 was over-expressed or knocked down. Meanwhile, EdU detection obtained the same results. These results suggest that PEAR1 exerts a positive regulatory effect on C2C12 cell proliferation.

As a member of the epidermal-growth-factor-like protein family, MEGF10 has been demonstrated to regulate the balance of proliferation and differentiation of MuSCs through the Notch signaling pathway [20]. The extracellular structure of PEAR1 is similar to Notch signaling pathway receptor Jagged1, and it influences differentiation through the Notch signaling pathway during the early differentiation process of murine hematopoietic stem cells [7]. This implied that PEAR1 could potentially act as a new ligand in Notch signaling pathway. During skeletal muscle development, Notch1 and Notch2 are recognized as receptor proteins that promote muscle growth and development [21]. The question arises of whether PEAR1 could function as a ligand for Notch1 or Notch2. To test this hypothesis, the present study conducted immunoprecipitation experiments to investigate the interaction between PEAR1 and Notch1 or Notch2. The results confirmed that PEAR1 can indeed interact with Notch1 and Notch2 separately, thus suggesting that PEAR1 has the potential to serve as a novel ligand in the Notch signaling pathway.

The immunoprecipitation experiment has already demonstrated that PEAR1 can interact with the Notch signaling pathway receptor proteins Notch1 and Notch2 individually. The question of whether PEAR1 can serve as a ligand for the Notch signaling pathway, thereby regulating its activity, becomes a pivotal aspect in uncovering how PEAR1 modulates C2C12 cell proliferation. Therefore, this study further performed overexpression and interference experiments with PEAR1, and Western blotting was used to assess the expression levels of the downstream marker molecule of the Notch signaling pathway, Hes1 (an important downstream effector molecule of the Notch signaling pathway). The results showed that upon overexpression or interference with PEAR1 expression, the expression level of Hes1 similarly increased or decreased. These findings indicate that during C2C12 cell proliferation, PEAR1 can function as a ligand for the Notch signaling pathway. It achieves this by interacting with the Notch1 and Notch2 receptors to modulate the activity of the Notch signaling pathway. To further confirm that PEAR1 regulates C2C12 cell proliferation through the Notch signaling pathway, the study simultaneously overexpressed PEAR1 and inhibited the Notch signaling pathway by adding the inhibitor RIN1 during C2C12 cell proliferation. Through Western blotting, the expression changes of relevant proteins were examined. The results showed that only during PEAR1 overexpression, the expression levels of the downstream marker molecule Hes1 and proliferation markers Pax7, CCNB1, and PCNA all increased. When RIN1 was added to inhibit the Notch signaling pathway, the expression levels of Hes1, Pax7, CCNB1, and PCNA significantly decreased, indicating that interference with the Notch signaling pathway suppressed cell proliferation. When both PEAR1 overexpression and RIN1 inhibition were combined, the expression levels of Hes1, Pax7, CCNB1, and PCNA all significantly decreased. These results collectively indicate that the C2C12 cell proliferation regulated by PEAR1 is dependent on the modulation of the Notch signaling pathway.

The aforementioned research has established that PEAR1 regulates cell proliferation through the Notch signaling pathway during the proliferation phase of C2C12 cells. However, whether PEAR1 possesses the same function in vivo remains unclear. Therefore, this study established a mouse post-injury regeneration model. Bupivacaine hydrochloride, a local anesthetic known for its neuro- and myotoxicity. It is typically injected into the target tissue to damage skeletal muscles. Typically, it is injected into the target tissue to damage skeletal muscles. A single small injection of bupivacaine hydrochloride can cause muscle fiber necrosis without affecting the activity of MuSCs. The muscle fiber injury stimulus can rapidly trigger satellite cells to repair the damaged tissue. HE staining results confirmed the successful establishment of the skeletal muscle post-injury regeneration model.

High expression of Pax7 is crucial for maintaining the quiescent state of MuSCs and plays a significant role in the self-stabilization of muscle tissue equilibrium [18]. On the other hand, MyoD is a determinant molecule that drives MuSCs towards myogenic differentiation [22]. In this experiment, both Pax7 and MyoD protein expression levels were found to be elevated on the first and third days after the injury, indicating the activation and extensive proliferation of MuSCs during these periods. Throughout the process of skeletal muscle post-injury regeneration, the expression patterns of PEAR1 and Hes1 were similar to those of Pax7 and MyoD proteins. This suggests that PEAR1 and the Notch signaling pathway may play significant regulatory roles in the proliferation of MuSCs. Additionally, research indicates that Hes1 maintains the quiescent state of MuSCs and the stability of muscle tissue by inhibiting MyoD expression [23]. Therefore, it is hypothesized that even when injury occurs, the activity of the Notch pathway remains at a certain level, promoting the activation and proliferation of quiescent MuSCs while preventing their excessive differentiation. At the peak of the most severe damage (on the third day), the Notch signaling pathway is activated, promoting extensive proliferation of MuSCs. Upon completion of repair, the Notch signaling pathway maintains a higher level, contributing to the self-stabilization of muscle tissue.

Both Notch receptor proteins, Notch1 and Notch2, demonstrate elevated expression levels during the process of skeletal muscle post-injury regeneration. This complexity highlights the role of the Notch signaling pathway in regulating organism activity. Due to the highly complex muscle microenvironment in which MuSCs reside [24], further research is needed to elucidate the relationship between PEAR1 and the Notch signaling pathway as well as their molecular mechanisms in regulating muscle satellite cell proliferation. This study employed immunohistochemical staining techniques to detect Pax7 expression, thereby locating MuSCs. Furthermore, the expression and localization of PEAR1, Notch1, and Notch2 were observed. The immunohistochemical results revealed the following: Pax7, as a marker molecule for MuSCs, is expressed at a high level at the damaged site when skeletal muscle is injured. On the third day of injury repair, Pax7-positive signals reached their peak, indicating extensive proliferation of MuSCs at this stage. In the subsequent phases of the regeneration, the intensity of Pax7-positive signals diminished, suggesting a decrease in satellite cell proliferation. During this phase, the predominant physiological activities at the injury site involve the differentiation and fusion of MuSCs. Only a small portion of MuSCs re-enter the cell cycle to become quiescent satellite cells, thus maintaining the stability of the satellite cell pool [25,26].

Furthermore, studies have indicated that specific activation of the Notch signaling pathway in mice leads to muscle cells dedifferentiating into Pax7-positive quiescent MuSCs [27]. The Notch signaling pathway plays critical roles in embryonic and post-natal skeletal muscle development. Meanwhile, numerous studies have indicated the significance of the Notch signaling pathway in regulating MuSCs proliferation and self-renewal, which plays important roles in skeletal muscle post-injury regeneration [28,29].

The activation of the Notch signaling pathway depends on the interaction between Notch ligand and receptors on adjacent cell membranes. In this study, PEAR1 may be involved in the injury and regeneration of mouse skeletal muscle as a novel ligand of the Notch signaling pathway, but its specific mechanism remains unclear. In future studies, we will conduct experiments in mice, such as gene knockout or adeno-associated virus (AAV) infection experiments, to further explore the role of PEAR1 in skeletal muscle post-injury regeneration and the molecular mechanism of PEAR1 in Notch signaling pathway. This is also a limitation of our current study.

## 5. Conclusions

This study demonstrated the molecular mechanism by which PEAR1, as a ligand for the Notch signaling pathway, regulates the proliferation of MuSCs through in vitro experiments and may play an important regulatory role in the proliferation of mouse skeletal muscle satellite cells through the construction of skeletal muscle post-injury regeneration model.

## Data Availability

Data are contained within the article.

## Supplementary Materials

The following supporting information can be downloaded at: https://www.mdpi.com/article/10.3390/biology13121063/s1.
