# Supplementary figures and images for: PEAR1 Promotes Myoblast Proliferation Through Notch Signaling Pathway
Source: Biology (Basel). 2024 Dec 19;13(12):1063. doi: 10.3390/biology13121063 (PMC11673774; doi:10.3390/biology13121063)

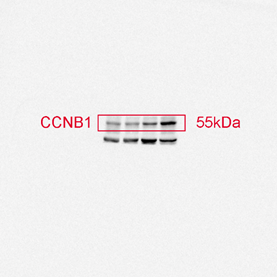

Supplement: Supplementary file 1 [file biology-13-01063-s001.zip › supplementary/Raw data for Western blotting/Figure 1/CCNB1.jpg]

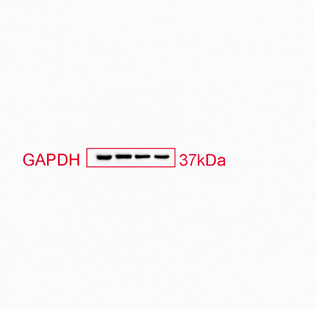

Supplement: Supplementary file 1 [file biology-13-01063-s001.zip › supplementary/Raw data for Western blotting/Figure 1/GAPDH.jpg]

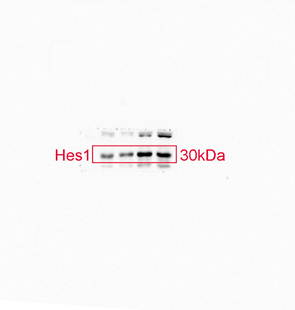

Supplement: Supplementary file 1 [file biology-13-01063-s001.zip › supplementary/Raw data for Western blotting/Figure 1/Hes1.jpg]

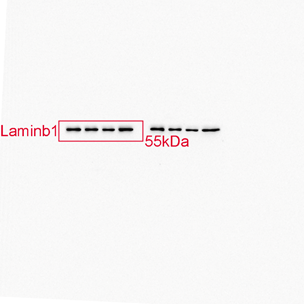

Supplement: Supplementary file 1 [file biology-13-01063-s001.zip › supplementary/Raw data for Western blotting/Figure 1/Laminb1.jpg]

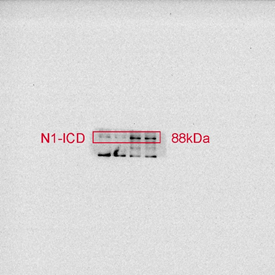

Supplement: Supplementary file 1 [file biology-13-01063-s001.zip › supplementary/Raw data for Western blotting/Figure 1/N1-ICD.jpg]

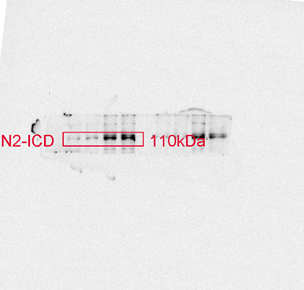

Supplement: Supplementary file 1 [file biology-13-01063-s001.zip › supplementary/Raw data for Western blotting/Figure 1/N2-ICD.jpg]

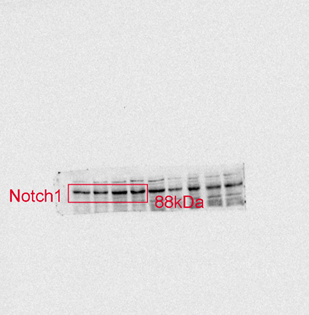

Supplement: Supplementary file 1 [file biology-13-01063-s001.zip › supplementary/Raw data for Western blotting/Figure 1/Notch1.jpg]

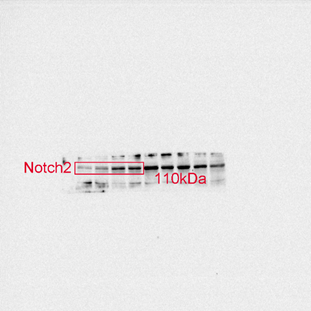

Supplement: Supplementary file 1 [file biology-13-01063-s001.zip › supplementary/Raw data for Western blotting/Figure 1/Notch2.jpg]

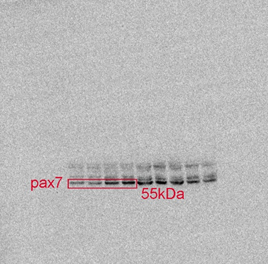

Supplement: Supplementary file 1 [file biology-13-01063-s001.zip › supplementary/Raw data for Western blotting/Figure 1/PAX7.jpg]

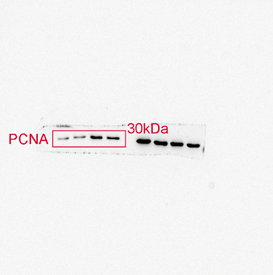

Supplement: Supplementary file 1 [file biology-13-01063-s001.zip › supplementary/Raw data for Western blotting/Figure 1/PCNA.jpg]

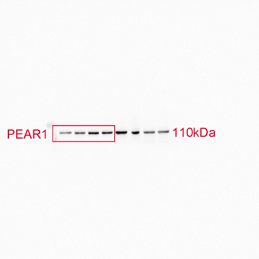

Supplement: Supplementary file 1 [file biology-13-01063-s001.zip › supplementary/Raw data for Western blotting/Figure 1/PEAR1.jpg]

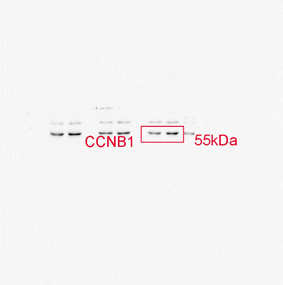

Supplement: Supplementary file 1 [file biology-13-01063-s001.zip › supplementary/Raw data for Western blotting/Figure 2A/CCNB1.jpg]

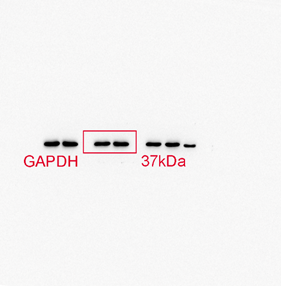

Supplement: Supplementary file 1 [file biology-13-01063-s001.zip › supplementary/Raw data for Western blotting/Figure 2A/GAPDH.jpg]

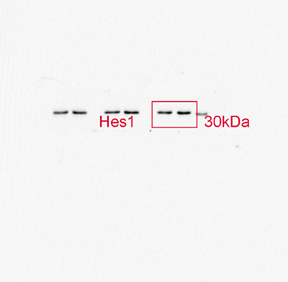

Supplement: Supplementary file 1 [file biology-13-01063-s001.zip › supplementary/Raw data for Western blotting/Figure 2A/Hes1.jpg]

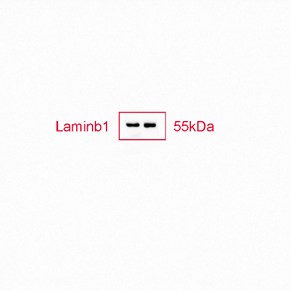

Supplement: Supplementary file 1 [file biology-13-01063-s001.zip › supplementary/Raw data for Western blotting/Figure 2A/Laminb1.jpg]

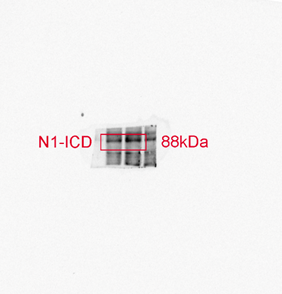

Supplement: Supplementary file 1 [file biology-13-01063-s001.zip › supplementary/Raw data for Western blotting/Figure 2A/N1-ICD.jpg]

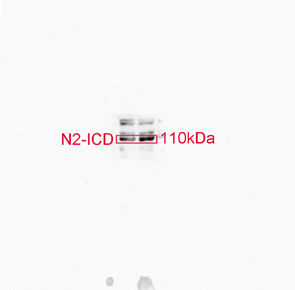

Supplement: Supplementary file 1 [file biology-13-01063-s001.zip › supplementary/Raw data for Western blotting/Figure 2A/N2-ICD.jpg]

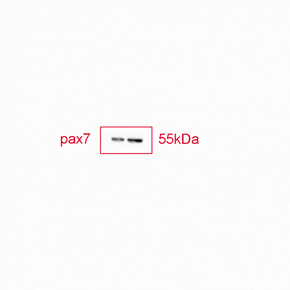

Supplement: Supplementary file 1 [file biology-13-01063-s001.zip › supplementary/Raw data for Western blotting/Figure 2A/PAX7.jpg]

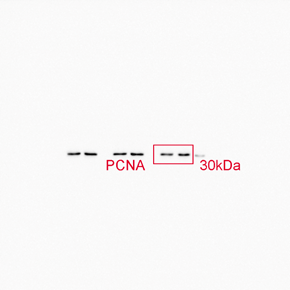

Supplement: Supplementary file 1 [file biology-13-01063-s001.zip › supplementary/Raw data for Western blotting/Figure 2A/PCNA.jpg]

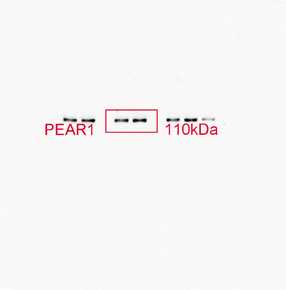

Supplement: Supplementary file 1 [file biology-13-01063-s001.zip › supplementary/Raw data for Western blotting/Figure 2A/PEAR1.jpg]

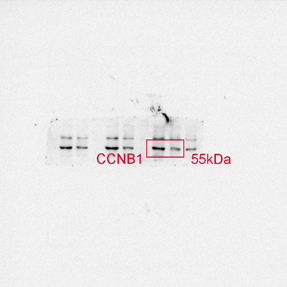

Supplement: Supplementary file 1 [file biology-13-01063-s001.zip › supplementary/Raw data for Western blotting/Figure 2C/CCNB1.jpg]

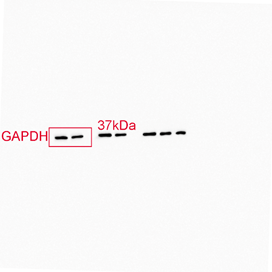

Supplement: Supplementary file 1 [file biology-13-01063-s001.zip › supplementary/Raw data for Western blotting/Figure 2C/GAPDH.jpg]

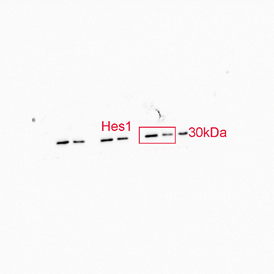

Supplement: Supplementary file 1 [file biology-13-01063-s001.zip › supplementary/Raw data for Western blotting/Figure 2C/Hes1.jpg]

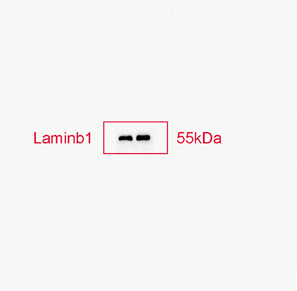

Supplement: Supplementary file 1 [file biology-13-01063-s001.zip › supplementary/Raw data for Western blotting/Figure 2C/Laminb1.jpg]

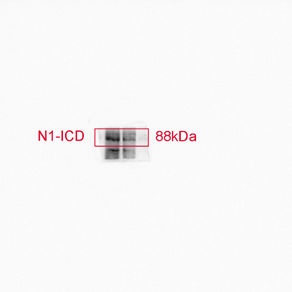

Supplement: Supplementary file 1 [file biology-13-01063-s001.zip › supplementary/Raw data for Western blotting/Figure 2C/N1-ICD.jpg]

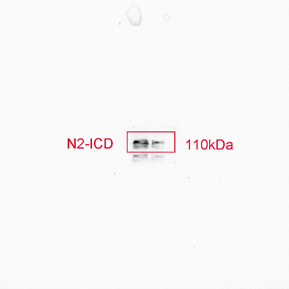

Supplement: Supplementary file 1 [file biology-13-01063-s001.zip › supplementary/Raw data for Western blotting/Figure 2C/N2-ICD.jpg]

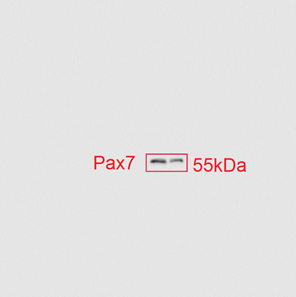

Supplement: Supplementary file 1 [file biology-13-01063-s001.zip › supplementary/Raw data for Western blotting/Figure 2C/PAX7.jpg]

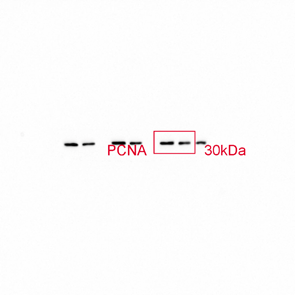

Supplement: Supplementary file 1 [file biology-13-01063-s001.zip › supplementary/Raw data for Western blotting/Figure 2C/PCNA.jpg]

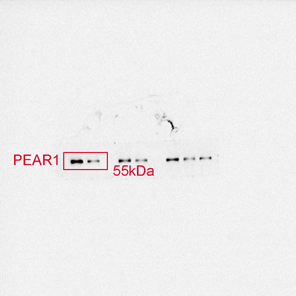

Supplement: Supplementary file 1 [file biology-13-01063-s001.zip › supplementary/Raw data for Western blotting/Figure 2C/PEAR1.jpg]

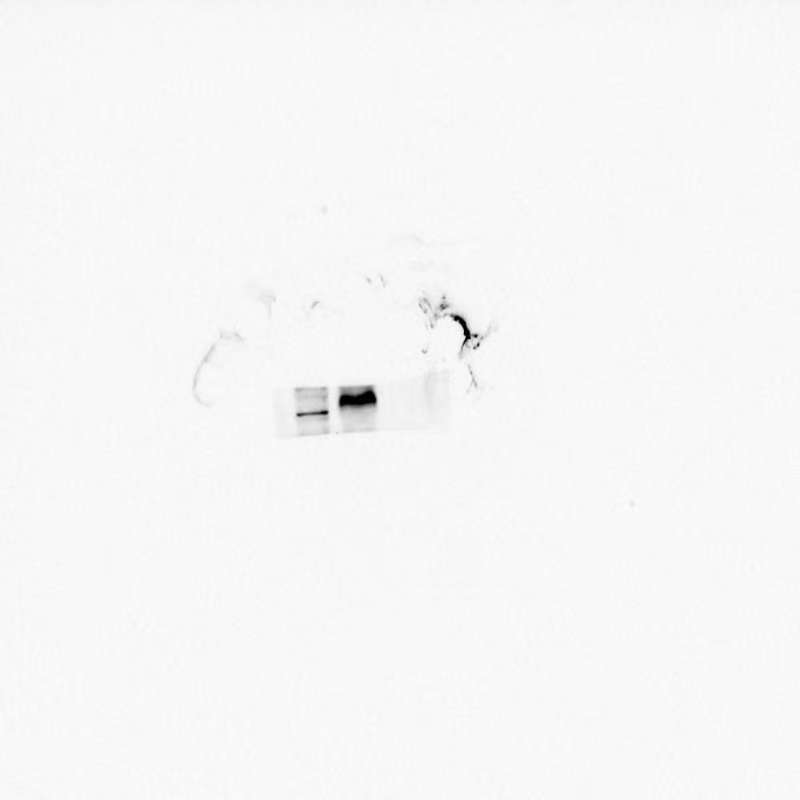

Supplement: Supplementary file 1 [file biology-13-01063-s001.zip › supplementary/Raw data for Western blotting/Figure 4/A Notch1.jpg]

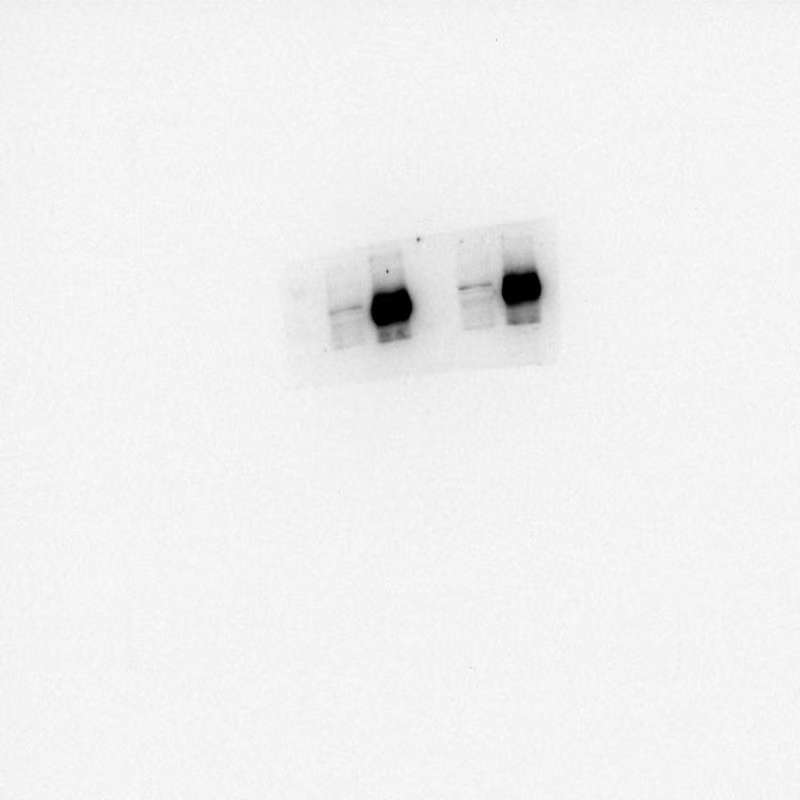

Supplement: Supplementary file 1 [file biology-13-01063-s001.zip › supplementary/Raw data for Western blotting/Figure 4/A PEAR1.jpg]

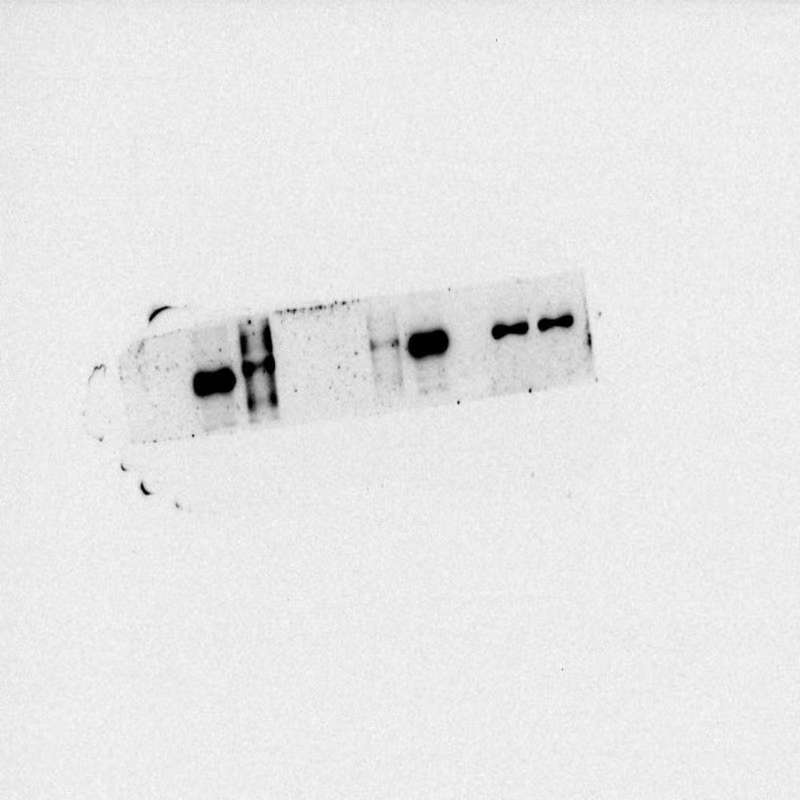

Supplement: Supplementary file 1 [file biology-13-01063-s001.zip › supplementary/Raw data for Western blotting/Figure 4/B Notch1.jpg]

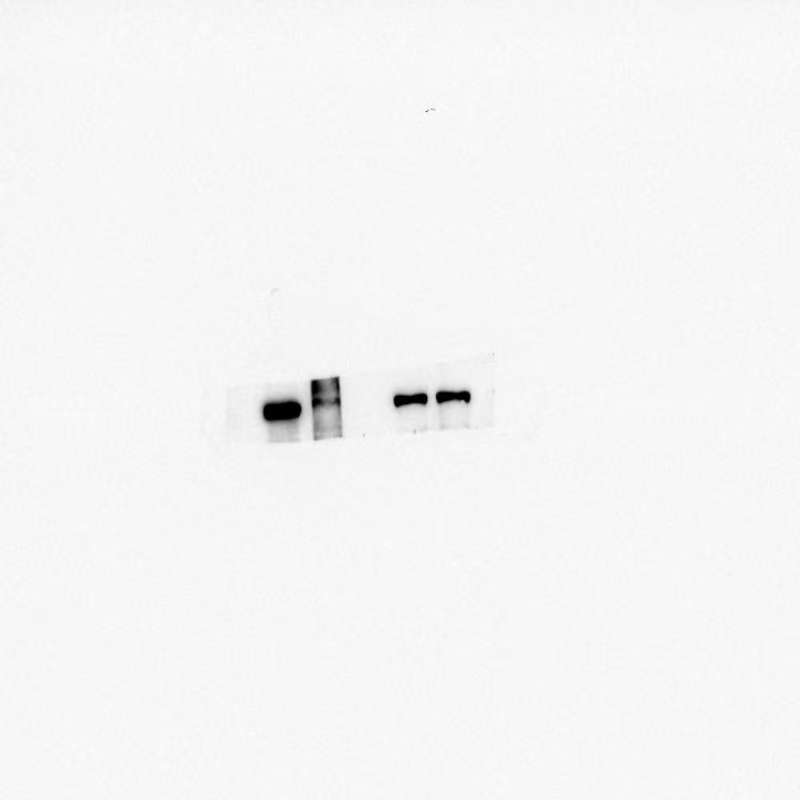

Supplement: Supplementary file 1 [file biology-13-01063-s001.zip › supplementary/Raw data for Western blotting/Figure 4/B PEAR1.jpg]

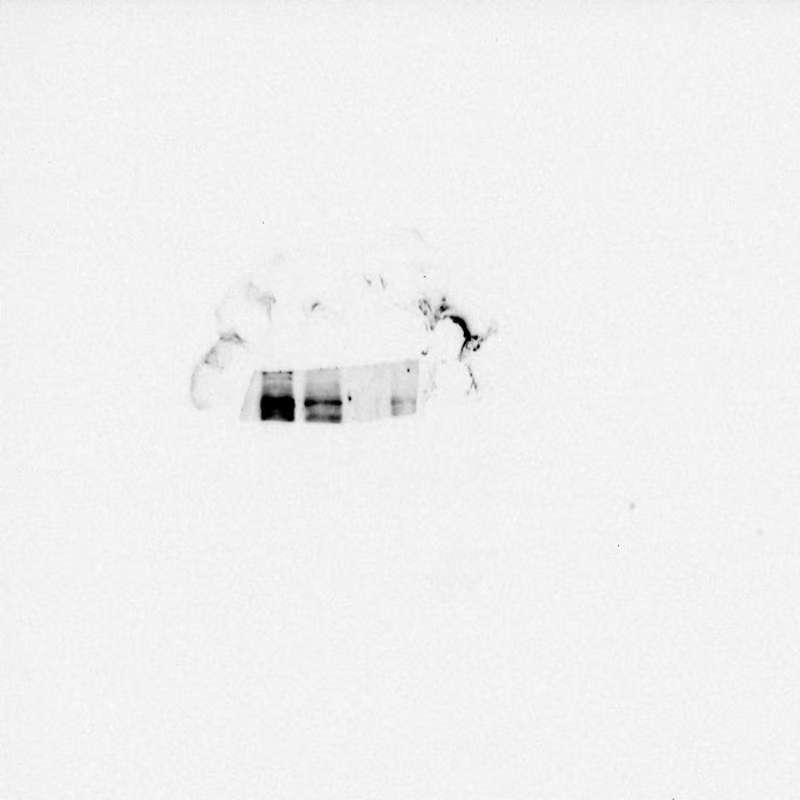

Supplement: Supplementary file 1 [file biology-13-01063-s001.zip › supplementary/Raw data for Western blotting/Figure 4/C Notch2.jpg]

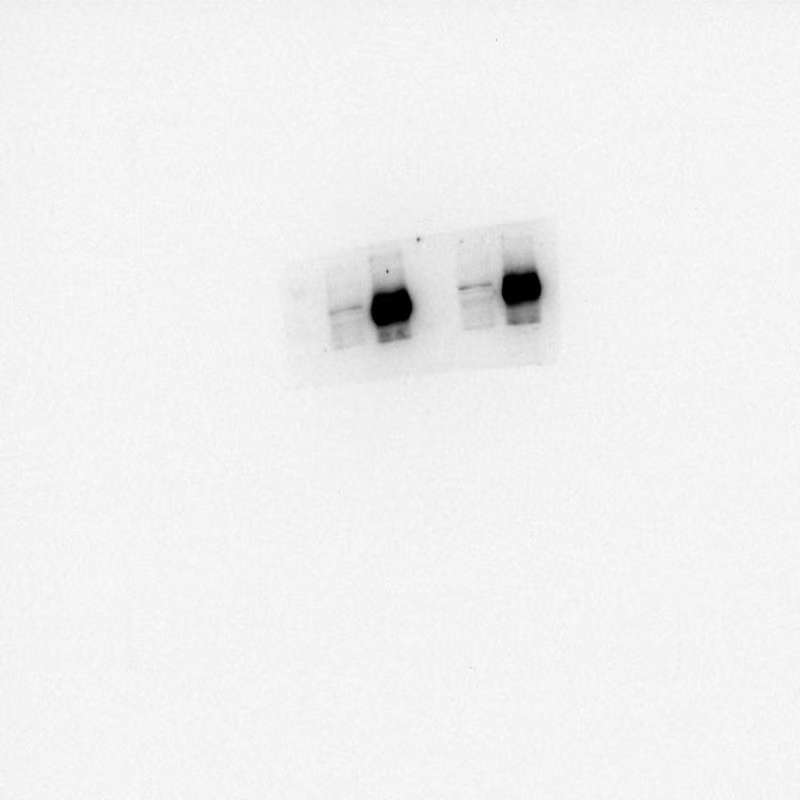

Supplement: Supplementary file 1 [file biology-13-01063-s001.zip › supplementary/Raw data for Western blotting/Figure 4/C PEAR1.jpg]

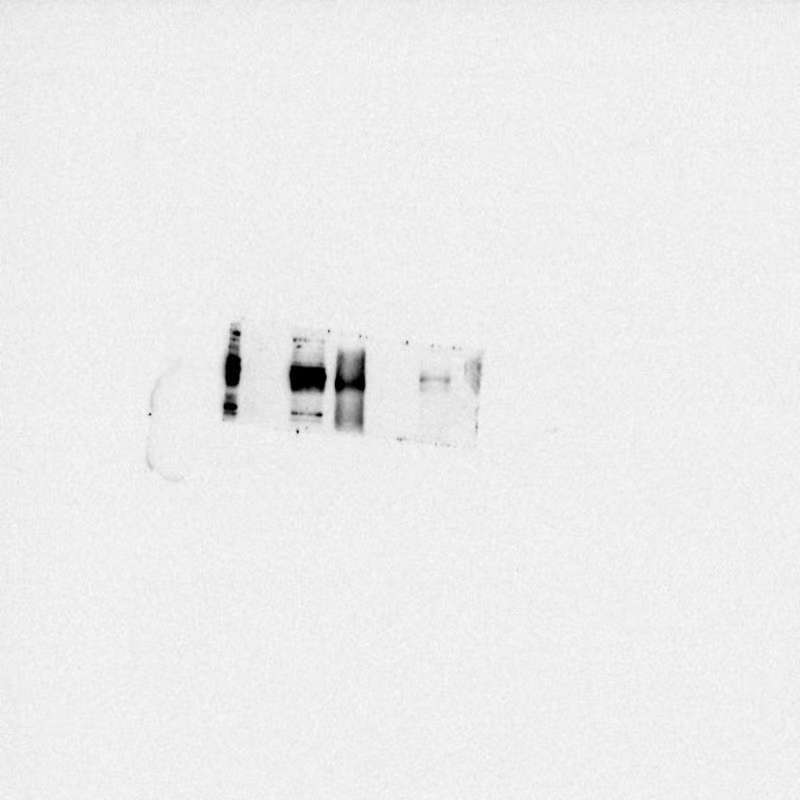

Supplement: Supplementary file 1 [file biology-13-01063-s001.zip › supplementary/Raw data for Western blotting/Figure 4/D Notch2.jpg]

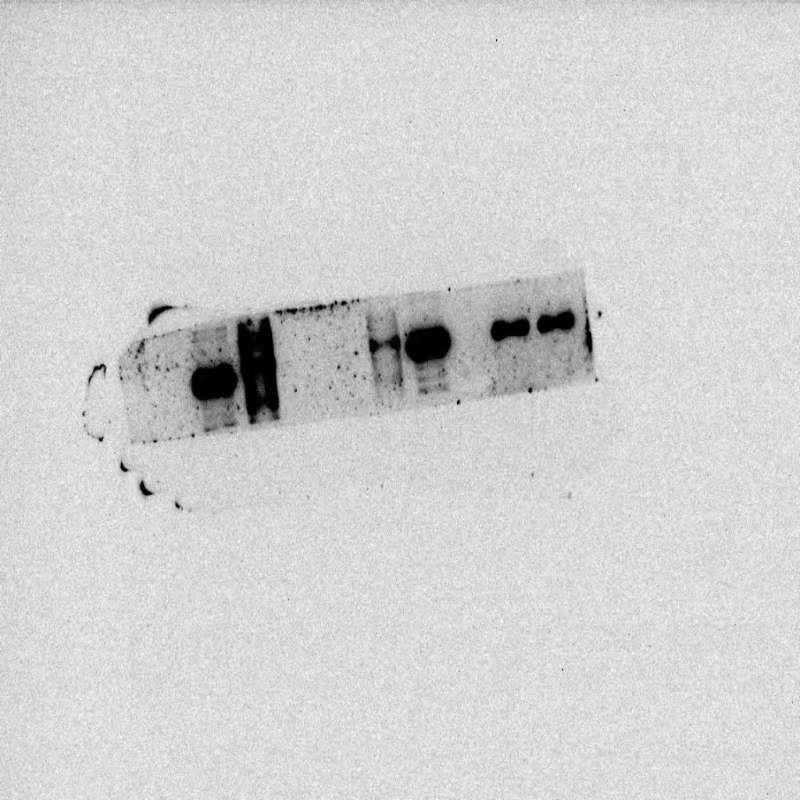

Supplement: Supplementary file 1 [file biology-13-01063-s001.zip › supplementary/Raw data for Western blotting/Figure 4/D PEAR1.jpg]

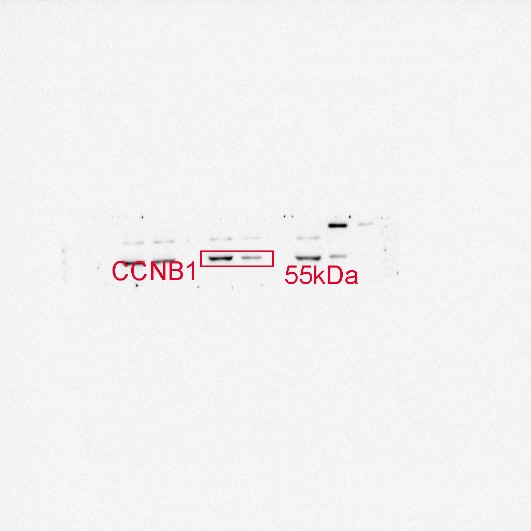

Supplement: Supplementary file 1 [file biology-13-01063-s001.zip › supplementary/Raw data for Western blotting/Figure 5A/ccnb1.jpg]

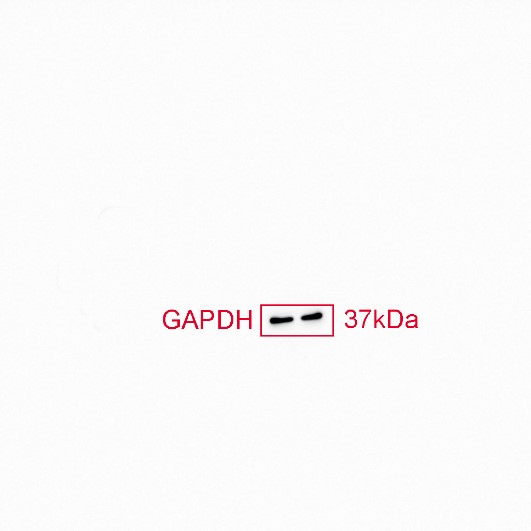

Supplement: Supplementary file 1 [file biology-13-01063-s001.zip › supplementary/Raw data for Western blotting/Figure 5A/gapdh.jpg]

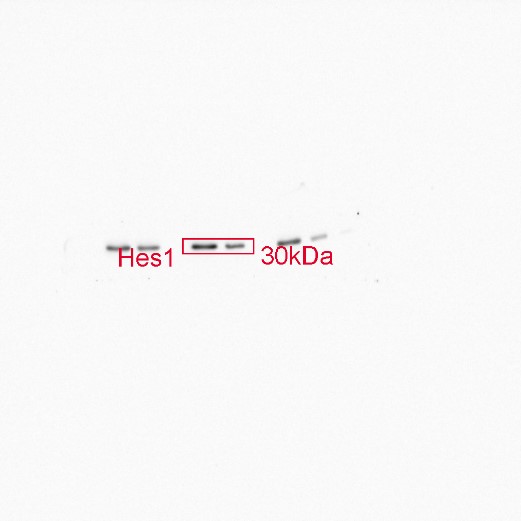

Supplement: Supplementary file 1 [file biology-13-01063-s001.zip › supplementary/Raw data for Western blotting/Figure 5A/Hes1.jpg]

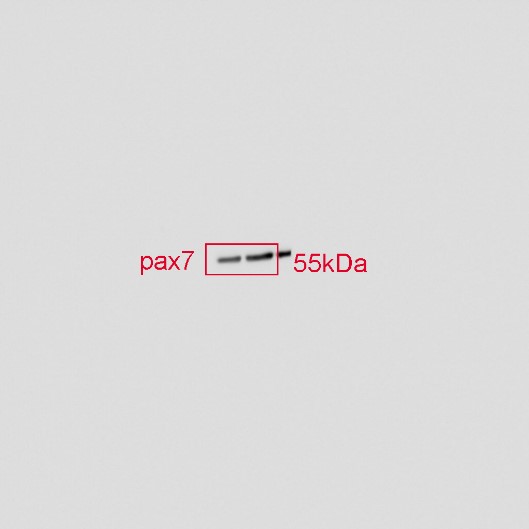

Supplement: Supplementary file 1 [file biology-13-01063-s001.zip › supplementary/Raw data for Western blotting/Figure 5A/pax7.jpg]

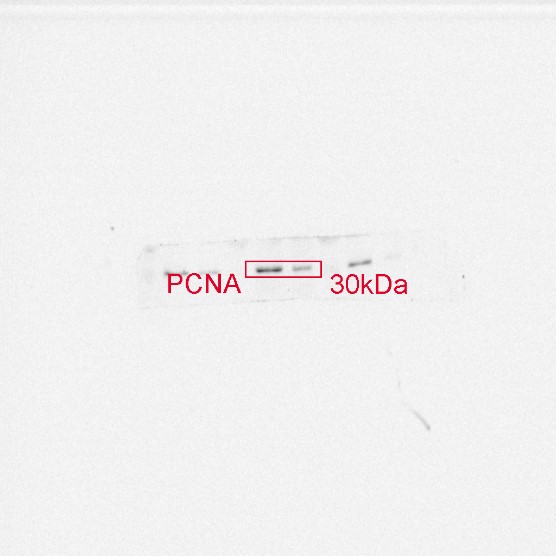

Supplement: Supplementary file 1 [file biology-13-01063-s001.zip › supplementary/Raw data for Western blotting/Figure 5A/pcna.jpg]

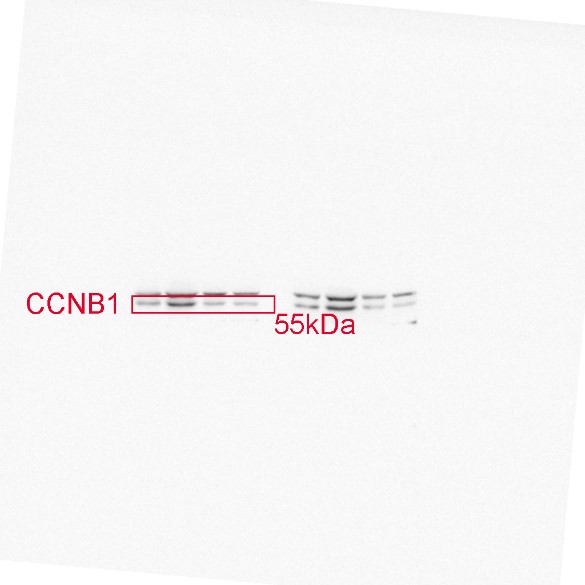

Supplement: Supplementary file 1 [file biology-13-01063-s001.zip › supplementary/Raw data for Western blotting/Figure 5H/ccnb1.jpg]

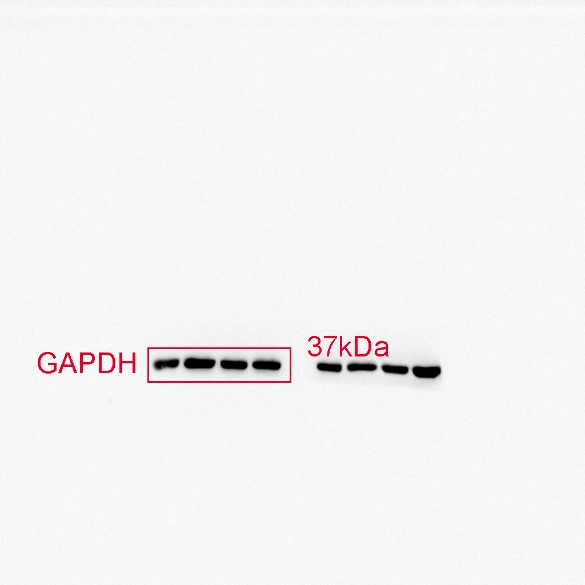

Supplement: Supplementary file 1 [file biology-13-01063-s001.zip › supplementary/Raw data for Western blotting/Figure 5H/gapdh1.jpg]

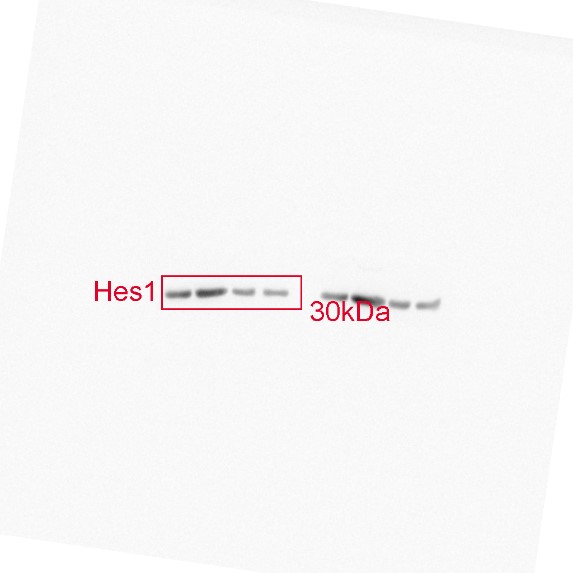

Supplement: Supplementary file 1 [file biology-13-01063-s001.zip › supplementary/Raw data for Western blotting/Figure 5H/hes1.jpg]

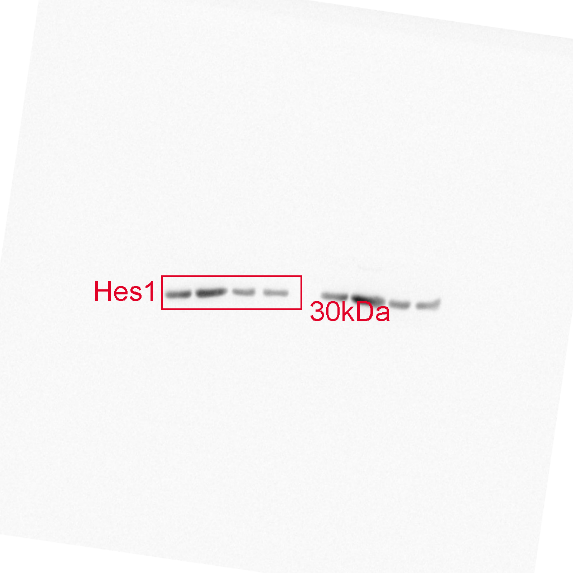

Supplement: Supplementary file 1 [file biology-13-01063-s001.zip › supplementary/Raw data for Western blotting/Figure 5H/hes1.tif]

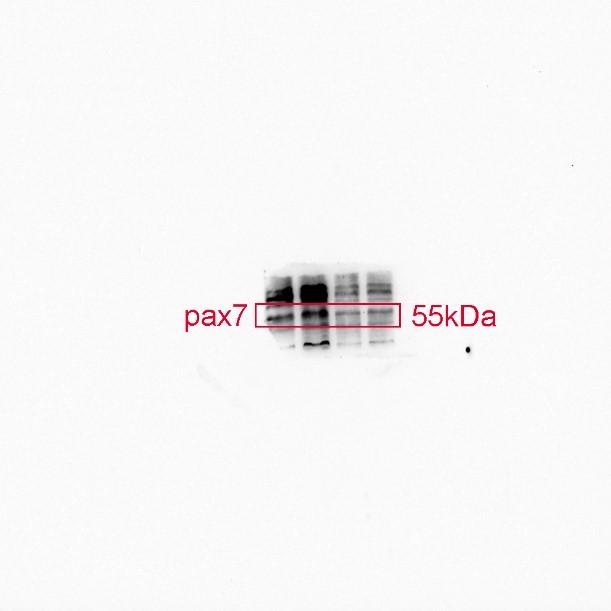

Supplement: Supplementary file 1 [file biology-13-01063-s001.zip › supplementary/Raw data for Western blotting/Figure 5H/pax7.jpg]

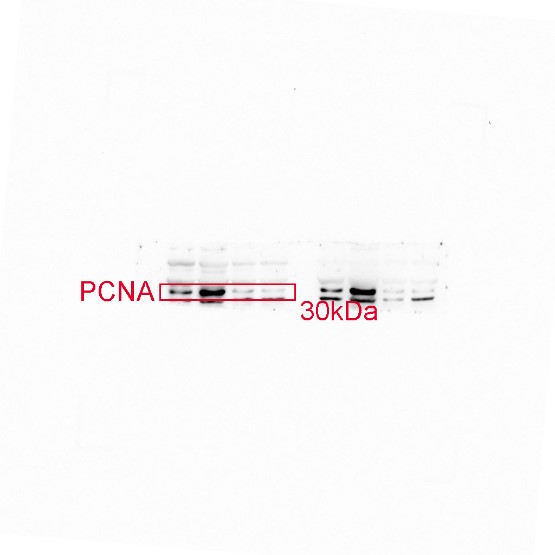

Supplement: Supplementary file 1 [file biology-13-01063-s001.zip › supplementary/Raw data for Western blotting/Figure 5H/pcna.jpg]

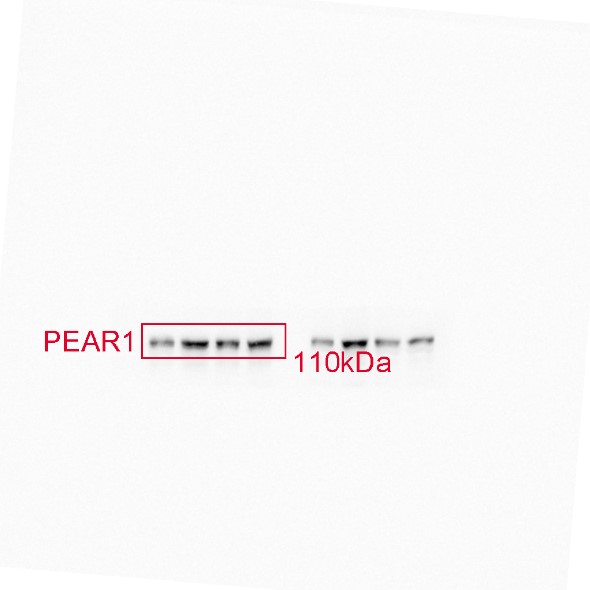

Supplement: Supplementary file 1 [file biology-13-01063-s001.zip › supplementary/Raw data for Western blotting/Figure 5H/pear1.jpg]

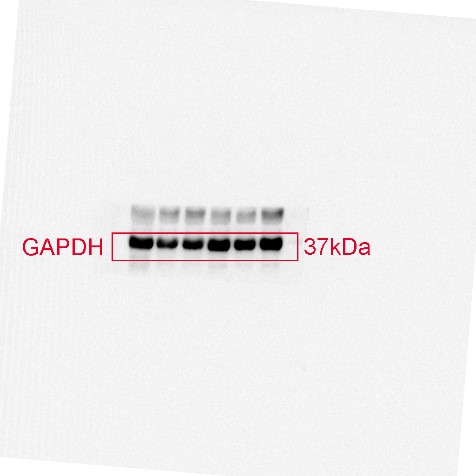

Supplement: Supplementary file 1 [file biology-13-01063-s001.zip › supplementary/Raw data for Western blotting/Figure 6B/gapdh.jpg]

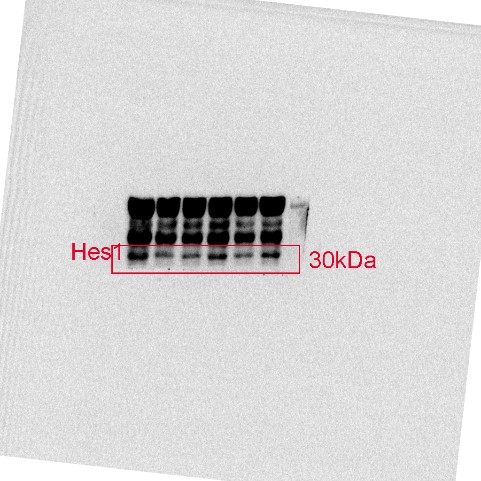

Supplement: Supplementary file 1 [file biology-13-01063-s001.zip › supplementary/Raw data for Western blotting/Figure 6B/hes1.jpg]

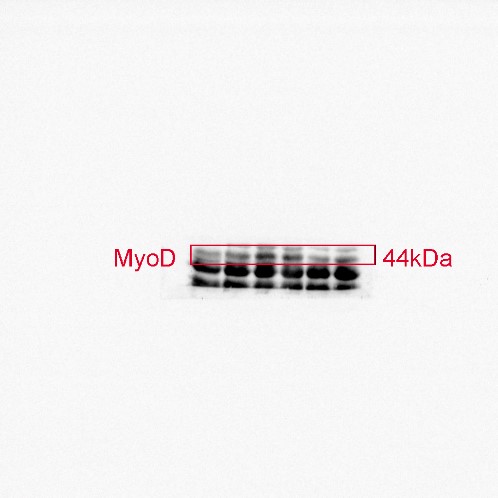

Supplement: Supplementary file 1 [file biology-13-01063-s001.zip › supplementary/Raw data for Western blotting/Figure 6B/myod.jpg]

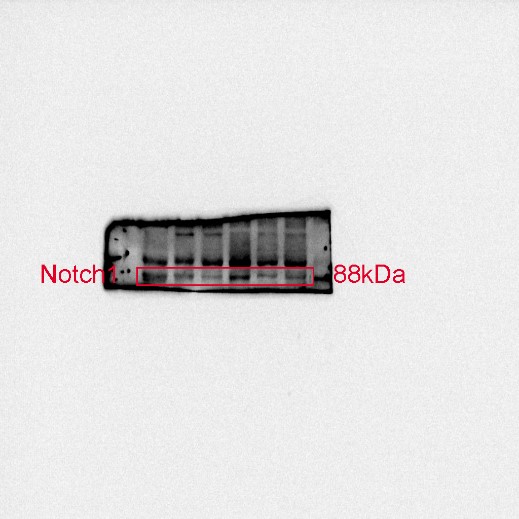

Supplement: Supplementary file 1 [file biology-13-01063-s001.zip › supplementary/Raw data for Western blotting/Figure 6B/notch1.jpg]

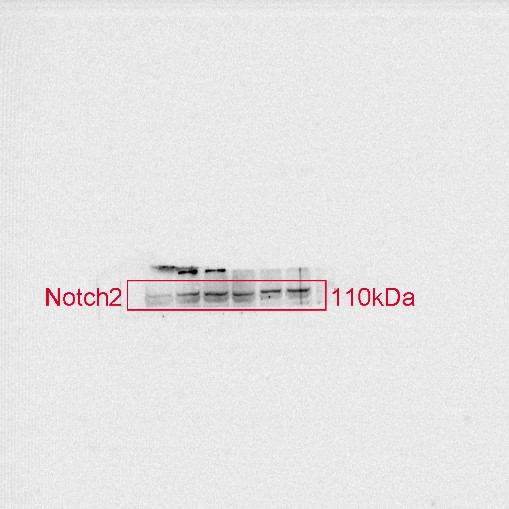

Supplement: Supplementary file 1 [file biology-13-01063-s001.zip › supplementary/Raw data for Western blotting/Figure 6B/notch2.jpg]

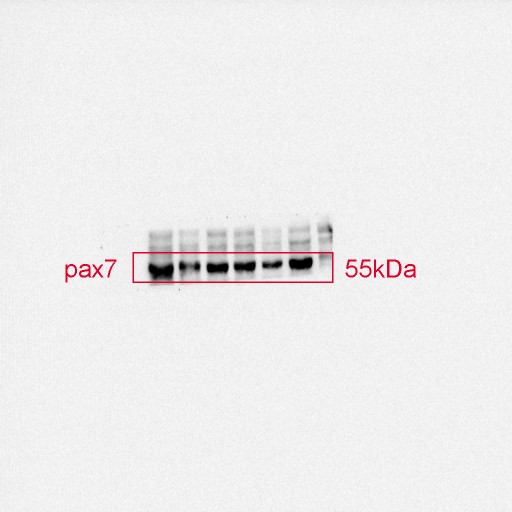

Supplement: Supplementary file 1 [file biology-13-01063-s001.zip › supplementary/Raw data for Western blotting/Figure 6B/pax7.jpg]

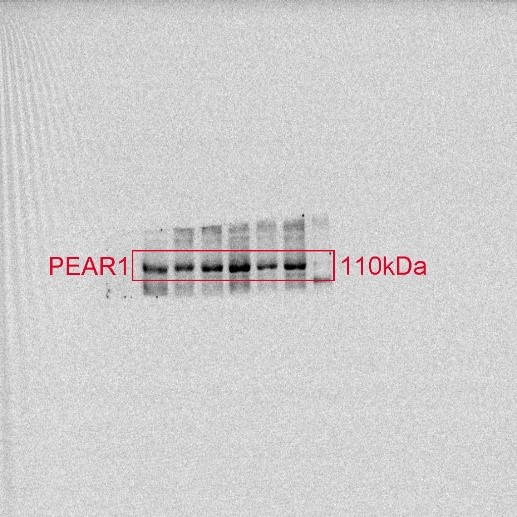

Supplement: Supplementary file 1 [file biology-13-01063-s001.zip › supplementary/Raw data for Western blotting/Figure 6B/pear1.jpg]
